# A Bifan Motif Shaped by ArsR1, ArsR2, and Their Cognate Promoters Frames Arsenic Tolerance of *Pseudomonas putida*

**DOI:** 10.3389/fmicb.2021.641440

**Published:** 2021-03-12

**Authors:** Gonzalo Durante-Rodríguez, David Páez-Espino, Víctor de Lorenzo

**Affiliations:** Systems Biology Department, Centro Nacional de Biotecnología-CSIC, Madrid, Spain

**Keywords:** arsenic, detoxification, *Pseudomonas putida*, cross-regulation, transcriptional factor, bifan motif

## Abstract

Prokaryotic tolerance to inorganic arsenic is a widespread trait habitually determined by operons encoding an As (III)-responsive repressor (ArsR), an As (V)-reductase (ArsC), and an As (III)-export pump (ArsB), often accompanied by other complementary genes. Enigmatically, the genomes of many environmental bacteria typically contain two or more copies of this basic genetic device *arsRBC*. To shed some light on the logic of such apparently unnecessary duplication(s) we have inspected the regulation—together and by separate—of the two *ars* clusters borne by the soil bacterium *Pseudomonas putida* strain KT2440, in particular the cross talk between the two repressors ArsR1/ArsR2 and the respective promoters. DNase I footprinting and gel retardation analyses of *Pars1* and *Pars2* with their matching regulators revealed non-identical binding sequences and interaction patterns for each of the systems. However, *in vitro* transcription experiments exposed that the repressors could downregulate each other’s promoters, albeit within a different set of parameters. The regulatory frame that emerges from these data corresponds to a particular type of bifan motif where all key interactions have a negative sign. The distinct regulatory architecture that stems from coexistence of various ArsR variants in the same cells could enter an adaptive advantage that favors the maintenance of the two proteins as separate repressors.

## Introduction

Various chemical species of arsenic pollute soil and water of many countries and cause serious environmental and health issues (Rosen, 1971; Páez-Espino et al., 2009) in a fashion that depends on the chemical species (+V, +III, 0, −III; Rosen, 2002; Oremland and Stolz, 2003). Arsenic is a very abundant metalloid that can be found widespread in the Earth crust. Different chemical species of the element originate both in natural sources (organic-rich or black shale, mineralized and mined areas, volcanogenic areas, and thermal springs) as well as the result of human activities (mining, waste processing, pesticides; Nordstrom, 2002). Virtually all microorganisms have evolved mechanisms for coping with arsenic toxicity (Páez-Espino et al., 2009) and some even use arsenate [AsO_4_] ^3–^ or arsenite [AsO_3_] ^3–^ as electron acceptor or donor (Tamaki and Frankenberger, 1992; Ahmann et al., 1994). Among the different bacterial strategies to deal with the toxic forms of arsenic the most common involves the so-called *ars* operon, the products of which bring about extrusion of As (III) and As (V) oxyanions out of the cells (Rosen, 1995; Xu et al., 1998; Oremland and Stolz, 2003; Páez-Espino et al., 2009). The core components of such operon include three genes: *arsR*, *arsB*, and *arsC*, that encode, respectively, a transcriptional regulator, an efflux pump of arsenite and an arsenate reductase responsible to catalyze the transformation of arsenate in arsenite (Xu et al., 1998). Although it is possible to find more genes in the *ars* operon depending on the bacterial species (*arsA*, *arsD*, *arsH*, and others; Suzuki et al., 1997, 1998; Silver, 1998; Páez-Espino et al., 2020) the most conserved are the three cited above. Genes encoding ArsR proteins belong to the family of ArsR-SmtB sensors, a widespread group of transcriptional factors characterized by the ability to respond to arsenic, antimony and bismuth. These regulators embody two distinct domains i.e., a DNA binding segment with a typical helix-turn-helix motif (HTH; Barbosa et al., 2007), and a ligand-docking amino acid sequence with a α3 helix signature, capable of binding arsenite (Busenlehner et al., 2003).

**FIGURE 1:**
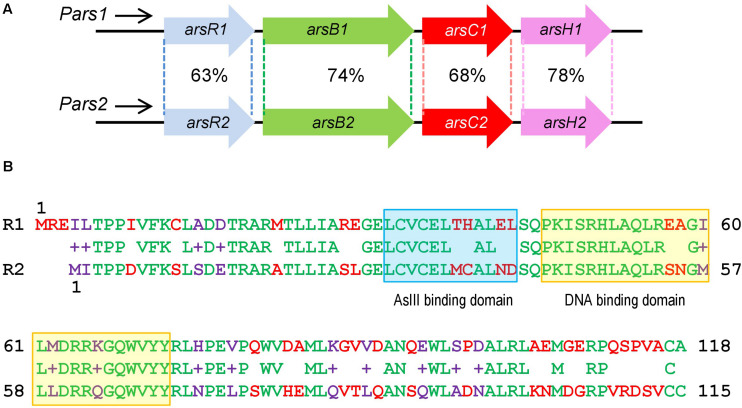
Similarities between *Pseudomonas putida* KT2440 *ars1* and *ars2* operons based on sequence identity and length of the alignments. **(A)** *P. putida* KT2440 *ars* operons description: Identity of amino acid sequence between the two *ars* operons. **(B)** Comparison of ArsR1 and ArsR2 amino acid sequence. The top amino acid sequence belongs to ArsR1 protein with 118 residues. The bottom belongs to ArsR2 protein with 115 residues. The middle belongs to the sequence alignment. The amino acids are highlighted in green (conserved), red (non-conserved), and purple (non-conserved but it shows functional equivalence). The As (III) binding domain is marked in a blue box and the DNA binding domain is marked in a yellow box.

As is the case with other bacteria, the soil ubiquitous and saprophytic bacterium *Pseudomonas putida* strain KT2440, a microorganism with a high adaptability to diverse environments and nutrients, has also an arsenic resistance system (Cánovas et al., 2003; Páez-Espino et al., 2009) which enables growth at very high concentrations of arsenate (in the range of 300 mM) and arsenite (in the range of 5 mM). However, unlike other microorganisms, the arsenic resistance phenotype of *P. putida* KT2440 stems not from one, but two chromosomally encoded *ars* operons. Such apparently redundant gene clusters have a DNA sequence identity between homologous genes around 68–78% (Figure 1A), while the corresponding primary proteins overlap by 73–87% (Páez-Espino et al., 2015). This seemingly superfluous duplication has been proposed to be both a way to reinforce the phenotype (Fernández et al., 2014) and a case of ecoparalogy (Sánchez-Pérez et al., 2008). This involves co-existence of both clusters for enabling the cognate products to run under different temperature conditions (Páez-Espino et al., 2015).

In this work, we have addressed some molecular details of the regulation of the two *ars* operons of *P. putida* in particular the organization and performance of the corresponding promoter regions. Specifically, the binding sites recognized by the regulators in their cognate promoters and the ensuing regulatory consequences have been sorted out. Moreover, we have studied the functionality of the ArsR variants in each operon and the role of arsenite and arsenate as transcriptional inducers. Finally, we provide evidence of cross-regulation between both operons, where ArsR1 and ArsR2 are able to bind and regulate reciprocally the expression of both promoter regions, *Pars1* and *Pars2*. The resulting regulatory architecture originates a typical bifan regulatory motif (Resendis-Antonio et al., 2005; Lipshtat et al., 2008) that is densely populated by a large number of side-connections among the basic components of the respective transcriptional nodes. On this basis, we entertain that the coexistence of two systems of As tolerance in *P. putida* is not a mere contingency in the evolutionary history of this bacterium, but an adaptive trait that has made this bacterium particularly resistant to the oxyanion.

## Materials and Methods

### Bacterial Strains, Plasmids, and Growth Conditions

*Escherichia coli* and *P. putida* KT2440 cells were grown at 37°C or 30°C, respectively, in Luria-Bertani (LB) medium (Sambrook and Russell, 1989). Experiments in Petri dishes were made with the same media added with 1.5% (w/v) agar. Where appropriate, antibiotics were added at the following concentrations: ampicillin (Ap, 150 μg/ml) and kanamycin (Km, 50 μg/ml). Where needed, the medium was added with filtered sodium arsenite (NaH_2_AsO_3_) or sodium arsenate (NaH_2_AsO_4_) from Sigma Aldrich Chemicals, as necessary for the experiment at stake.

### Overproduction and Purification of 6His-ArsR1 and 6His-ArsR2 Proteins

To construct recombinant plasmids pQE32-ArsR1 and pQE32-ArsR2, 373-bp and 364-bp PCR-amplified fragments that include the *arsR1* and *arsR2* genes were obtained by using *P. putida* KT2440 genomic DNA as template and the pair of oligonucleotides 5′ ArsR1-His (5′-CGGGATCCTTCGAGAAATACTGACTCCCCCCA-3′, engineered *Bam*HI is underlined) and 3′ ArsR1-His (5′-GGGAAGCTTTCAAGCACAAGCAACAGGGCT-3′, engineered *Hin*dIII is underlined), and 5′ ArsR2-His (5′-CGGGATCCTTATCACACCGCCCGATGTCTT-3′, engineered *Bam*HI is underlined) and 3′ ArsR2-His (5′-GGGAAGCTTTCAGCAGCAGACGGAATCACG-3′, engineered *Hin*dIII is underlined), respectively. The *arsR1* and *arsR2* fragments were digested with *Bam*HI and *Hin*dIII restriction enzymes and ligated to the *Bam*HI/*Hin*dIII double-digested pQE32 6His-tag vector rendering plasmids pQE32-ArsR1 and pQE32-ArsR2, respectively. The recombinant plasmids pQE32-ArsR1 and pQE32-ArsR2 express under control of the *T5* promoter and two *lac* operator boxes the genes encoding the proteins 6His-ArsR1 and 6His-ArsR2 that carry 12 amino acids (MRGSHHHHHHGIL) fused to their terminus. The 6His-ArsR1 and 6His-ArsR2 proteins were overproduced in *E. coli* M15 strain harboring plasmids pQE32-ArsR1 and pQE32-ArsR2, respectively, and the pREP4 plasmid that produces the LacI repressor to strictly control gene expression from pQE32 derivatives in presence of isopropyl-1-thio-β-D-galactopyranoside (IPTG). *E. coli* M15 (pREP4, pQE32-ArsR1) and *E. coli* M15 (pREP4, pQE32-ArsR2) cells were grown at 37°C in 200 ml of ampicillin- and kanamycin-containing LB medium until the cultures reached mid-exponential growth phase. Overexpression of the His-tagged proteins was then induced during 5 h by the addition of IPTG 0.5 mM. Cells were harvested at 4°C, resuspended in 20 ml of lysis buffer (50 mM NaH_2_PO_4_, 300 mM KCl, 20 mM imidazole, pH 8.0) and disrupted by passage through a French press (Aminco Corp.) operated at a pressure of 20,000 p.s.i. The cell lysate was centrifuged at 26,000 × *g* for 30 min at 4°C. The clear supernatant fluid was carefully decanted and applied to nickel-nitrilotriacetic acid-agarose columns (Qiagen). Columns were then washed at 4°C with 50 volumes of washing buffer (50 mM NaH_2_PO_4_, 300 mM KCl, 50 mM imidazole, pH 8.0), and the 6His-ArsR1 and 6His-ArsR2 proteins were eluted by using elution buffer (50 mM NaH_2_PO_4_, 300 mM KCl, 500 mM imidazole, pH 8.0). The purified proteins were dialyzed at 4°C in dialyzing buffer (20 mM NaH_2_PO_4_, 100 mM KCl, 10% glycerol, 2 mM β-mercaptoethanol, pH 8.0) and stored at −20°C.

### Molecular Biology Techniques

Recombinant DNA methods were carried out as published (Miller, 1972; Sambrook and Russell, 1989). Plasmid DNA was prepared with High Pure Plasmid Isolation Kit (Roche Applied Science). DNA fragments were purified with Gene Clean Turbo (Q-BIOgene). Oligonucleotides were supplied by Sigma. All cloned inserts and DNA fragments were confirmed by DNA sequencing with an ABI Prism 377 automated DNA sequencer (Applied Biosystems). Transformation of *E. coli* was carried out by using the RbCl method or by electroporation (Gene Pulser, Bio-Rad) (Miller, 1972). Transformation of *P. putida* KT2440 was carried out by electroporation (Choi et al., 2006). Proteins were analyzed in a SDS-PAGE system (Sambrook and Russell, 1989) with a 15% acrylamide/bisacrylamide (37.5:1) gel cast in a Mini-PROTEAN 3 Cell (Bio-Rad), following standard protocols. Proteins were resuspended in a denaturing buffer containing 2% sodium dodecyl sulfate (SDS), 5% glycerol, 60 mM Tris–HCl pH 6.8, 1% β-mercaptoethanol and 0.005% bromophenol blue, and boiled for 10 min prior to loading. Gels were stained with a 0.05% solution of Coomassie R-250 blue in methanol 50% and acetic acid 10%, and de-stained in 10% methanol with 7% acetic acid.

### Sequence Data Analyses

For bioinformatic inspection of genes and regulatory regions of interest we employed the BioEdit Sequence Alignment Editor (Hall, 1999) and the ApE-A plasmid Editor v.1.17 (Copyright^©^ 2003–2008, M. Wayne David). The BLAST platform (Altschul et al., 1997) was used for studying similarity/identity of sequences. The amino acid sequences of the open reading frames were compared with those present in databases using the TBLASTN algorithm at the NCBI server^1^. Nucleotides and proteins alignments were done with ALIGN (Wilbur and Lipman, 1983) and CLUSTALW (Thompson et al., 1994), respectively, in the BioEdit editor.

### Analytical Ultracentrifugation

The oligomerization state of 6His-ArsR1 and 6His-ArsR2 was examined in an XLI analytical ultracentrifuge (Beckman-Coulter) equipped with a UV-visible absorbance detection system. Sedimentation velocity experiments were performed in an An-50Ti rotor at 20°C and 48,000 rpm loaded with samples of 6His-ArsR1 or 6His-ArsR2 at a concentration of 42 μM and 36 μM, respectively, prepared in 20 mM NaH_2_PO_4_, 100 mM KCl, 2 mM β-mercaptoethanol, pH 8.0 with or without 2 mM of arsenite. Sedimentation profiles were recorded every 3 min at 285 nm. The corresponding sedimentation coefficient distributions c(s) were calculated by least squares boundary modeling of sedimentation velocity data using SEDFIT v12.1 software (Schuck, 2000). Experimental sedimentation coefficients were corrected to standard solvent conditions (pure water, 20°C, and infinite dilution) using the SEDNTERP program (Laue et al., 1992) for generating the corresponding standard values (S20, W). Sedimentation equilibrium experiments were made in the same instrument under identical 6His-ArsR1 or 6His-ArsR2 concentrations and buffer conditions as the sedimentation velocity assays before. In this case, measurements were taken at 11,000 and 14,000 rpm using short columns with 100 μl of protein sample. After the equilibrium scans, a high-speed centrifugation run (43,000 rpm) was done to estimate the corresponding baseline offsets. The corresponding buoyant signal average molecular weights of the samples were determined by fitting the experimental data to the equation that characterizes the equilibrium gradient of an ideally sedimenting solute using Hetero-Analysis software (Cole, 2004). The absolute molecular weight (MW) of 6His-ArsR1 and 6His-ArsR2 was determined from the experimental buoyant molecular weight values using the partial specific volume (0.733 ml/g) calculated from the amino acid composition using SEDNTERP (Laue et al., 1992).

### *Pars1-lacZ* and *Pars2-lacZ* Transcriptional Fusions and β-Galactosidase Assays

In order to generate DNA segments containing all regulatory elements driving transcription of the *Pars1* and *Pars2* promoters we utilized the *Pars1-lacZ* and *Pars2-lacZ* fusions in the plasmids pSEVA225-*Pars1* and pSEVA225-*Pars2* described by Páez-Espino et al. (2015). For determination of promoter activity, plasmids pSEVA225-*Pars1* and pSEVA225-*Pars2* were passed to *P. putida* KT2440 (wt) as indicated. The transformed strains were grown at 30°C in LB medium in presence of increasing concentrations of arsenite (from 0 to 20 mM) until the cultures reached the stationary phase. At that point, accumulation of β-galactosidase was measured in permeabilized cells as described by Miller (1972).

### Gel Retardation Assays

To prepare a DNA fragment suitable for gel retardation experiments a 309-bp and 299-bp sequences containing the *Pars1* and *Pars2* promoters, respectively, were amplified using *P. putida* KT2440 genomic DNA as template, and the pair of oligonucleotides 5′ *Pars1*-Eco (see above) and 3′ *Pars1*-Bam (5′-AGAGGATCCATCAGCAGGGTCAT-3′, engineered *Bam*HI is underlined), and 5′ *Pars2*-Eco (5′-GCGAATTCATGTTGGCATCTCG-3′, engineered *Eco*RI is underlined) and 3′ *Pars2*-Bam (5′-AGAGGATCCTGGCGATAAGCAGA-3′, engineered *Bam*HI is underlined). The resulting DNA products were digested with *Eco*RI restriction enzyme and 3’ end-labeled by filling the overhanging end of the cleaved site with [α-^32^P]-dATP and Klenow DNA polymerase (Sambrook and Russell, 1989). Binding reactions were performed in 10 μl of TRRG buffer (20 mM Tris–HCl pH 7.5, 50 mM KCl, 2 mM β-mercaptoethanol, 10% v/v glycerol) containing 0.3 nM of end-labeled *Pars1* or *Pars2* probes, 5 mg of BSA, 25 μg/ml herring sperm (competitor) DNA and increasing amounts of 6His-ArsR1 or 6His-ArsR2 proteins ranging 5–2,000 nM. Samples where incubated for 20 min at 30°C and electrophoresed in a 5% non-denaturing polyacrylamide gel prepared in 0.5x TBE buffer (Sambrook and Russell, 1989). DNA band shifts were observed by autoradiography of the dried gels on X-ray film (Konica Minolta).

### DNase I Footprinting Assays

The DNA fragments used for DNase I footprinting assays were the same *Pars1* and *Pars2* probes as that reported for the gel retardation assays (see above). For the experiment, the reaction mixture contained 2 nM DNA probe (*Pars1* or *Pars2*), 1 mg/ml BSA and purified protein in 15 μl of TRRG buffer (see above). This mixture was incubated for 20 min at 37°C, after which 3 μl (0.05 enzyme units) of DNase I (Amersham Biosciences) (prepared in 10 mM CaCl_2_, 10 mM MgCl_2_, 125 mM KCl and 10 mM Tris–HCl, pH 7.5) were added, and the incubation was continued at 37°C for 20 s. The reaction was stopped by addition of 180 μl of solution containing 0.4 M sodium acetate, 2.5 mM EDTA, 50 μg/ml calf thymus DNA and 0.3 μg/ml glycogen, pH 8.9. After phenol extraction, DNA fragments were analyzed as described previously (Durante-Rodríguez et al., 2006). A + G Maxam and Gilbert reactions (Maxam and Gilbert, 1980) were carried out with the same fragments and loaded on the gels along with the footprinting samples. The gels were dried on Whatman 3MM paper and exposed to Hyperfilm MP (Amersham Biosciences).

### *In vitro* Transcription (IVT) Experiments

Transcription assays were performed as published previously (Arce-Rodríguez et al., 2012). Supercoiled templates bearing the *Pars1* and *Pars2* promoters were constructed as follows. 267-bp segments of genomic DNA of *P. putida* KT2440 containing the corresponding promoter sequences were amplified with primers 5′ *Pars1* (5′-CGGGATCCTGATCGGTACCAAGCAATCGG-3′, engineered *Bam*HI is underlined)/3′ *Pars1* (5′-CGGAATTCAAACGATGGGGGGAGTCAGTATT-3′, engineered *Eco*RI is underlined), and 5′ *Pars2* (5′-CGGGATCCATGTTGGCATCTCGGTTATGAGC-3′, engineered *Bam*HI is underlined)/3′ *Pars2* (5′- CGGAATTCCAGAGAGGCTTTTGAAGACATCG-3′, engineered *Eco*RI is underlined), respectively. The resulting *Pars1* and *Pars2* fragments were digested with *Bam*HI and *Eco*RI restriction enzymes to generate cohesive ends and ligated to the *Bam*HI/*Eco*RI double-digested pJCD vector, thereby rendering plasmids pJCD-*Pars1* and pJCD-*Pars2*, which were employed in all subsequent IVT experiments following procedures described in detail previously (Durante-Rodríguez et al., 2008). In brief, IVT mixtures were set in 50 μl volumes containing 50 mM Tris–HCl pH 7.5, 50 mM KCl, 10 mM MgCl_2_, 0.1 mM BSA, 10 mM dithiothreitol, 1 mM EDTA, 30 nM of purified *P. putida* RNA polymerase holoenzyme prepared as described (Johansson et al., 2008), 5 nM pJCD-*Pars1* or pJCD-*Pars2* and 6His-ArsR1 or 6His-ArsR2 proteins ranging from 20 to 500 nM as specified. Where indicated, such a mixture was pre-incubated at 30°C for 10 min with sodium arsenite (NaH_2_AsO_3_) ranging 0.1–2 mM or 2 mM sodium arsenate (NaH_2_AsO_4_) prior to transcription start. The reactions were initiated by adding cold ATP, CTP and GTP 500 μM (each) and UTP 50 μM premixed with 2.5 μCi of [α-^32^P]-UTP (3000 mCi mmol^–1^). Following a 15 min incubation of the samples at 30°C, transcription was halted with 50 μl of a STOP mixture of 50 mM EDTA, 350 mM NaCl and 0.5 mg ml^–1^ carrier tRNA, pH 7.0. mRNA was precipitated with absolute ethanol at −20°C and resuspended in loading buffer containing 7 M urea, 1 mM EDTA, 0.6 M glycerol, 0.9 mM bromophenol blue and 1.1 mM xylene cyanol, pH 7.5. The resulting samples were electrophoresed on a denaturing 7 M urea – 4% polyacrylamide gel, and visualized by autoradiography (Durante-Rodríguez et al., 2008).

## Results

### *arsR1* and *arsR2* Encode Transcriptional Regulators of *ars* Genes in *P. putida* KT2440

**FIGURE 2:**
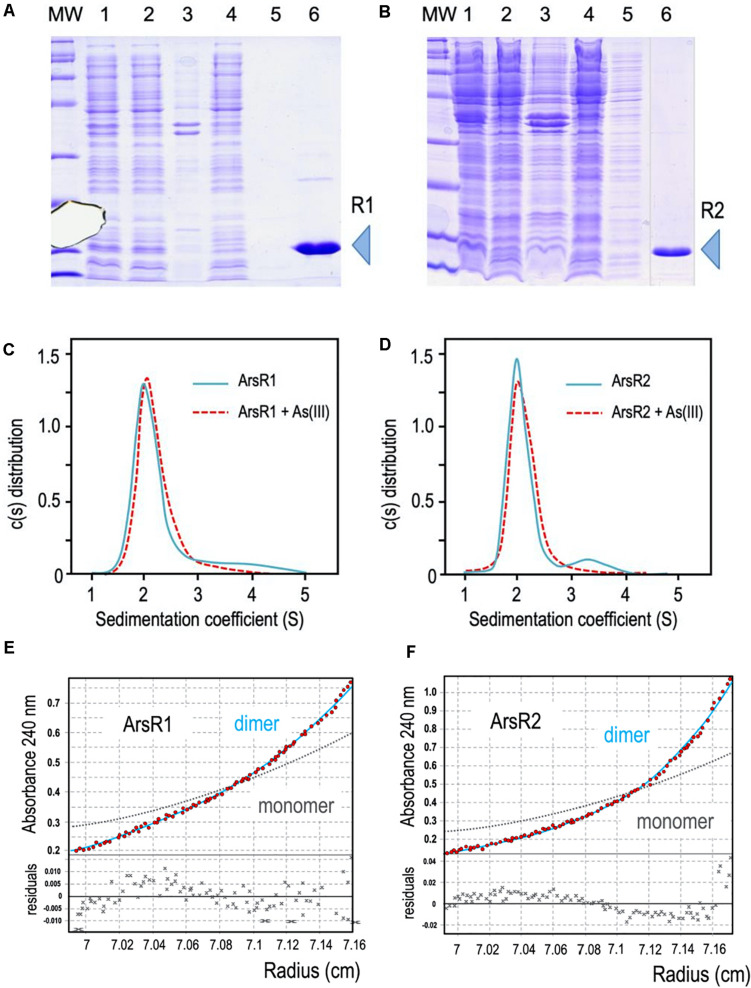
**(A,B)** 6His-ArsR1 and 6His-ArsR2 expression and purification. ArsR1 and ArsR2 proteins were cloned in a pQE32 expression vector to express with a 6His tag (see section “Materials and Methods”). In both gels, MW is the molecular weight marker, lane 1, cells after expression; lane 2, supernatant after break the cells; lane 3, pellet after break the cells; lane 4, flow through after pass the supernatant by the purification column; lane 5, sample after washing the column; lane 6, elution of the purification column with imidazole (R1 and R2 point out the purified proteins ArsR1 and ArsR2, respectively). Samples were loaded in a 15% denaturing PAGE stained with Coomassie blue as shown. Note that the image of sample 6 comes from the same gel but has been pasted next to lane 5 to avoid an empty lane in between. **(C,D)** Oligomerization state of ArsR1 and ArsR2. Plotted data correspond to the sedimentation coefficient distribution obtained in sedimentation velocity experiments with purified 6His-ArsR1 or 6His-ArsR2 (blue continuous line) and 6His-ArsR1 or 6His-ArsR2 bound with As (III) (red dotted line). The predominant fraction of the ArsR1 or ArsR2 species (93.5%) presented both a sedimentation coefficient of 2.1 ± 0.1 S. This value corresponds to a molecular mass compatible with the theoretical values of the proteins (confirmed by sedimentation equilibrium experiments, see text), which is consistent with a dimeric form of the proteins ArsR1 (29.5 kDa) and ArsR2 (29.1 kDa). Addition of As (III) doesn’t vary the sedimentation coefficient values of ArsR1 or ArsR2, keeping the dimeric state. **(E,F)** Study of the oligomerization state of the ArsR1 and ArsR2 proteins in solution. Sedimentation equilibrium data (*red dots*) and the best fit analysis assuming a theoretical protein dimer (*solid blue line*) and monomer (*broken gray line*) species. The *lower panel* shows residuals between estimated values and experimental data for protein dimer.

As described above, *P. putida* KT2440 possesses two different *ars* operons with the same structure and a high identity degree, composed of the genes *arsR*, *arsB*, *arsC*, and *arsH* (Cánovas et al., 2003; Páez-Espino et al., 2009; Figure 1A). Both clusters *ars1* and *ars2* contain functional *arsB* and *arsC* genes directly involved in detoxification of inorganic As species along with variants of an additional gene *arsH*. This last gene encodes an oxidoreductase that relieves oxidative stress caused by exposure to the oxyanion (Páez-Espino et al., 2020). By similarity with other systems, the *arsR* genes determine the transcriptional regulators of the operons. *arsR1* and *arsR2* genes share a 69% sequence identity and encode proteins of 118 and 115 amino acids, respectively, showing a 63% of identity between their primary sequences (Figure 1B; Páez-Espino et al., 2015). At the same time, they both exhibit significant similarity with transcriptional regulators of the SmtB/ArsR family (Busenlehner et al., 2003). It was possible to find the typical conserved motifs of this family within their amino acid sequence: an arsenite binding domain and a DNA binding domain (Figure 1B). To study in more detail the regulatory role of these transcriptional regulators, we purified ArsR1 and ArsR2 for running the *in vitro* assays shown below. To this end, the *arsR1* and *arsR2* genes were cloned into the pQE32 vector as detailed in the section “Materials and Methods.” Purification was done in a Ni-NTA column and eluted with imidazole, ultimately obtaining proteins high-purity 6His-ArsR1 (Figure 2A) and 6His-ArsR2 (Figure 2B).

To study the native conformation of the ArsR1 and ArsR2 regulators, we performed analytical ultracentrifugation experiments with the purified proteins. Sedimentation velocity experiments were carried out at different concentrations of regulators (5 to 20 μM) and analyzed in terms of distribution of sedimentation coefficients, allowing an evaluation of protein homogeneity and self-association. Figures 2C,D show the sedimentation velocity data for 20 μM of 6His-ArsR1 and 6His-ArsR2 in solution, demonstrating that under these conditions both proteins sediment as a unique species with an *s* value of 2.1 ± 0.1 S. The molecular weight of the 2.1 S species measured by sedimentation equilibrium was compatible with the mass of a dimer (6His-ArsR1 and 6His-ArsR2 monomers have a theoretical mass of 14,869 and 14,519 Da, respectively). Since reduced As (III) is the only species of the oxyanion that can be in the cytoplasm, we next tested whether exposure to arsenite had any effect on the aggregation state of either protein. For this, the same sedimentation velocity experiments were repeated with both proteins in the presence of the oxyanion. The data for 20 μM of 6His-ArsR1 and 6His-ArsR2 in solution in presence of 2 mM of arsenite overlapped with the same experiment before in absence of arsenite (Figures 2C,D). Both proteins kept the dimer state in presence of arsenite. This means that whatever regulatory effect As (III) may have on ArsR proteins, it does not alter the dimerization state—but plausibly affects the conformation of an already stable dimer. This altogether agrees with the default dimeric state known in many regulators of the SmtB/ArsR family (Busenlehner et al., 2003).

**FIGURE 3:**
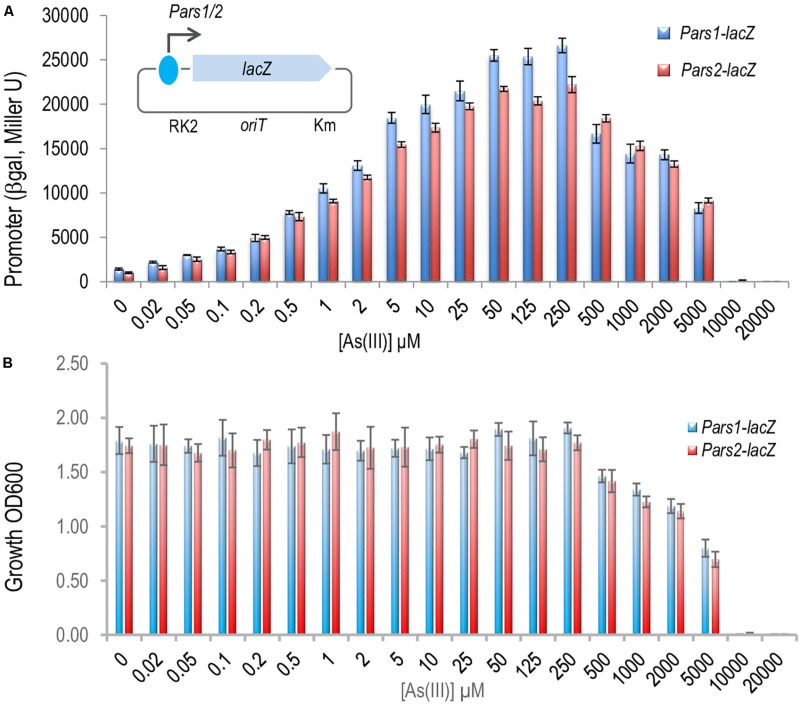
*In vivo* effect of the arsenite on the regulation of the *Pars1* and *Pars2* promoters. **(A)** *Pseudomonas putida* KT2440 (containing a transcriptional fusions *Pars1*-lacZ or *Pars2*-lacZ in the plasmids pSEVA225-*Pars1* or pSEVA225-*Pars2*, respectively; see section “Materials and Methods”) was grown for 24 h at 30°C in LB medium with increasing concentrations of arsenite as indicated until the mid-exponential culture phase. β-galactosidase activity is expressed in Miller units. Results from three independent experiments (*n* = 3) and errors bars are shown. **(B)** Toxic effect of arsenite in *P. putida* growth. *P. putida* KT2440 (containing the same transcriptional fusion plasmids) was grown for 24 h at 30°C in LB medium with increasing concentrations of arsenite as before. The cell growth was measured by spectrophotometry at 600 nm. Results from three independent experiments (*n* = 3) and errors bars are shown.

Once the ArsR proteins were at hand we set out to dissect the regulation of the system by studying the interaction between ArsR1 or ArsR2 and their respective promoters *Pars1* and *Pars2*. To secure that the DNA segments used in the experiments below captured all the regulatory sequences involved in transcriptional control, we exploited reporter plasmids pSEVA225-*Pars1* and pSEVA225-*Pars2* (Páez-Espino et al., 2015). They bear a 208 bp region, which spanned -182 to + 26 nt in respect to the transcription origin (estimated from transcriptomic data; Kim et al., 2013), ending just before the predicted RBS sequence of the *arsR1* and *arsR2* structural genes upstream of a promoterless *lacZ* reporter gene in a low copy number vector. These plasmids were separately transformed in *P. putida* KT2440, induced with As (III) and β-galactosidase accumulation recorded after 24 h of exposure to a range of arsenite concentrations. The data shown in Figure 3A indicated that the DNA inserts borne by the reporter plasmids endowed As (III)-responsive regulation to the reporter fusion, that the induction kinetics and that the associated parameters were very similar. One important detail is that maximum induction was reached by arsenite concentrations in the range of 250 μM—after which reporter readout started to go down, surely because of excessive toxicity of the added effector (Figure 3B). When the same experiments were repeated placing pSEVA225-*Pars1* and pSEVA225-*Pars2* in a double *ΔarsR1 ΔasrR2* mutant of *P. putida* KT2440 in both *arsR1* and *arsR2* genes, we obtained a constitutive expression of the cognate promoter in the absence of arsenite of ∼ 30,000 Miller units independent of the arsenite concentration (data not shown). Although these figures come from *in vivo* experiments, it suggested that interaction of arsenite with the transcriptional machinery is effective in the low micromolar range of the oxyanion. Note that the *in vivo* concentration to bring about half-induction was as low as 2–5 mM, that is lower than reported *in vitro* with purified proteins (∼30 mM; Fernández et al., 2016). This is likely to reflect the differences between intracellular and extracellular concentrations of the anion in the native cellular context caused by membrane-associated pumps and perhaps some intracellular channeling. It did not escape our notice either that when fully induced or when placed in a strain without the repressors, both *ars* promoters were very strong for a low-copy number (∼ 1–2 per cell) vector (Jahn et al., 2016). This is an interesting piece of data for future developments of the system as a device for biosensing purposes (Fernández et al., 2014) or heterologous gene expression.

### ArsR1 and ArsR2 Recognize and Bind a Specific SmtB/ArsR Consensus Box in *Pars1* and *Pars2* Promoters

**FIGURE 4:**
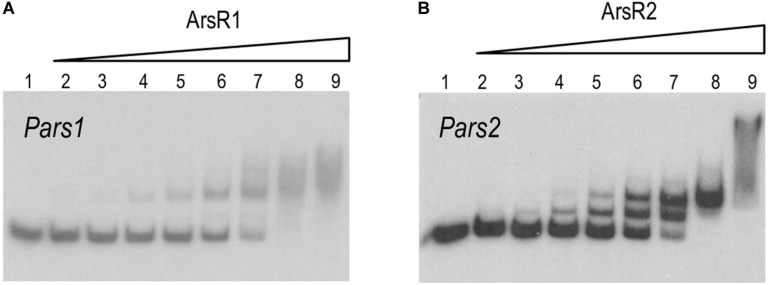
**(A,B)** *In vitro* binding of 6His-ArsR1 and 6His-ArsR2 proteins to the *Pars* promoters. Gel retardation analyses were performed as indicated in section “Materials and Methods.” In both gels, lane 1 shows the free *Pars1* or *Pars2* probes, respectively; lanes 2 to 9 show retardation assay of *Pars1* containing 5, 10, 25, 50, 100, 200, 500, and 1,000 nM of purified 6His-ArsR1 protein and retardation assay of *Pars2* containing 10, 25, 50, 100, 200, 500, 1,000, and 2,000 nM of purified 6His-ArsR2 protein.

Inspection of the *Pars1* and *Pars2* promoter regions of the reporter plasmids of Figure 3 revealed the presence of sequences 5′-CATATTCGAATAGTCATATATTCGGA-3′ and 5′-CACATATGGAAATACGTATATTCGGT-3′ overlapping, respectively, the −10 motif of each of the putative promoters (see below). Both DNA segments are very similar to the consensus sequence bound by repressors of the SmtB/ArsR family of proteins (Busenlehner et al., 2003), suggesting that they could be the binding regions for ArsR1 and ArsR2 proteins. To study these possible interactions, the purified 6His-ArsR1 and 6His-ArsR2 (see above) were mixed *in vitro* with the DNA fragments corresponding to *Pars1* and *Pars2* promoters and subjected to gel retardation assays. The 350 nt and 390 nt DNA probes with the *Pars1* and *Pars2* sequences encompassed nucleotides −300 to +50 and −340 to +50 in respect to transcription initiation, respectively. Both 6His-ArsR1 and 6His-ArsR2 were able to retard specifically migration of the *Pars1* and *Pars2* probes in a protein concentration-dependent manner (Figure 4) with a gross K_*D*_ of the DNA–protein interaction in the range of 200 nM for ArsR1 and 500 nM for ArsR2 on *Pars1* and *Pars2* promoters, respectively. Moreover, the proteins ArsR1 and ArsR2 showed non-identical extra retardation bands in the EMSA assays and their number, mobility and relative abundance did vary in each case. Since the methodology did not resolve the stoichiometry of the complexes, these bands were not trivial to interpret. Given that the protein seems to be always in a dimeric form, possibilities included binding 1 or 2 dimers and/or bending of the cognate DNAs around them. We cannot distinguish these possibilities with the available data. Yet, given that neither the DNA sequences of the respective promoters nor the proteins are identical, it cannot come as surprise that the details of the interactions are not the same either. In any case, specific binding of 6His-ArsR1 and 6His-ArsR2 to the *Pars1* and *Pars2* promoters was accredited by the fact that it was competed out by unlabeled probes with the same sequence—while it was not affected by adding a cold heterologous sequence (data not shown).

**FIGURE 5:**
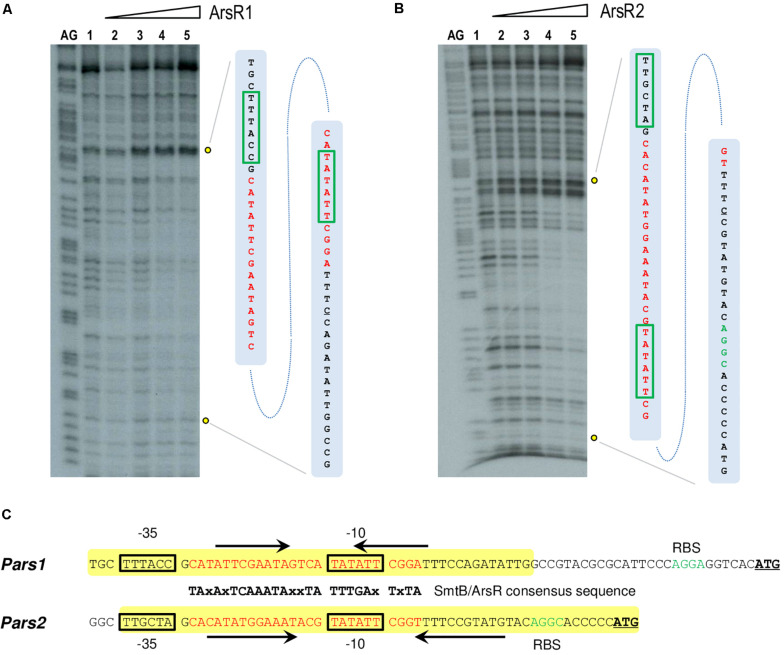
**(A,B)** DNase I footprinting analysis of the interaction of purified 6His-ArsR1 and 6His-ArsR2 with the *Pars1* and *Pars2* promoter regions, respectively. The DNase I footprinting experiments were carried out using the *Pars1* and *Pars2* probes labeled as indicated in section “Materials and Methods.” Lanes AG show the AG Maxam and Gilbert sequencing reactions. Both lanes 1 show a footprinting assay in the absence of proteins. Lanes 2 to 5 show footprinting assays containing 25, 50, 100, and 250 nM purified 6His-ArsR1 or 6His-ArsR2, respectively. An expanded view of the promoters regions protected by purified 6His-ArsR1 and 6His-ArsR2 is shown at the right of each figure. The ArsR binding sites are marked in red. The –10 and –35 boxes are also underlined in green. **(C)** Promoter sequences at the upstream region of the *ars1* and *ars2* genomic regions. The –35 and –10 motifs typical of σ^70^ promoters are in black boxes. The sequences encompassing in each case the operator regions based on the consensus target sites for repressors of the SmtB/ArsR family (Busenlehner et al., 2003) are indicated in red along with their cognate palindromic sequences (marked by black arrows). The yellow box shows the protected region of DNA observed in the footprinting assay. The ribosomal binding sites (RBS) are marked in green. The leading codon of each *arsR* gene in either operon is underlined in bold.

To gain some insight into the differential binding shown in Figure 4, the specific operators of each of the ArsR variants in their respective promoters were identified with DNase I footprinting assays. Figures 5A,B show that the 6His-ArsR1 and 6His-ArsR2 proteins protected a DNA region spanning from position −39 to +14 and −36 to +23 in respect to the predicted transcription start site of both promoters. As marked in Figure 5C, the protected region of each promoter contains partial palindromic sequences that are almost identical to the consensus SmtB/ArsR binding sequence mentioned before (Busenlehner et al., 2003), thereby confirming such regions as the *bona fide* ArsR operators at the *Pars1* and *Pars2* promoters. A closer inspection of the footprints suggested that in the case of Ars1, bands of lanes 4 and 5 seemed to be protected simultaneously. But in the case of Ars2, the upper part of the footprinted bands appeared to be more protected than the lower part. While the value of these observation is limited, it could reflect also the differences in the interactions exposed by the retardation experiments of Figure 4. Yet, in both instances, location of the ArsR binding sites overlapping the −10 box fitted the typical position of a repressor exerting its regulatory action by preventing binding of the σ^70^-RNA polymerase −10 box and therefore, blocking transcription initiation. To verify this prediction and gain an insight on the parameters ruling transcriptional control we next set out to reproduce the behavior of *Pars1* and *Pars2* promoters *in vivo* (Figure 3) in an *in vitro* system with purified components.

### ArsR1 and ArsR2 Mediate Repression of Their Respective Promoters *Pars1* and *Pars2*

**FIGURE 6:**
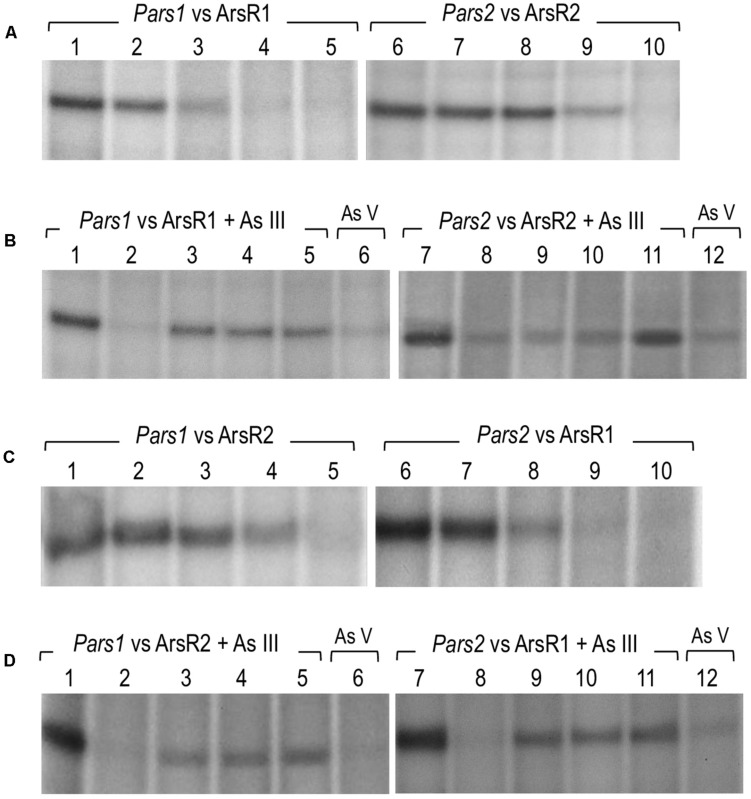
*In vitro* transcription showing the activity of the *Pars1* and *Pars2* promoters. Multiple-round transcription reactions were carried out by using plasmid pJCD harboring *Pars1* and *Pars2* promoter’s templates, as indicated, that produce an mRNA of 205 and 184 nucleotides, respectively. All the *in vitro* transcription reactions were performed with 40 nM *Pseudomonas putida* RNAP. **(A)** Effect of 6His-ArsR1 and 6His-ArsR2 proteins on *Pars1* and *Pars2* promoters, respectively. Lanes 1 to 5 were loaded with 0, 20, 50, 100, and 200 nM of 6His-ArsR1, and lanes 6 to 10 were loaded with 0, 20, 50, 100, and 500 nM of 6His-ArsR2. **(B)** Effect of As (III) and As (V) addition on the action of 6His-ArsR1 and 6His-ArsR2 on *Pars1* and *Pars2* promoters. Lanes 1 and 7 were loaded without any protein. Lanes 2 to 6 and 8 to 12 were performed with 100 nM of 6His-ArsR1 and 500 nM of 6His-ArsR2, respectively. Lanes 2 to 5 and 8 to 11 were performed with 0, 0.1, 0.5, and 2 mM of arsenite (AsIII), respectively. Lanes 6 and 12 correspond to samples with 2 mM of arsenate (AsV). **(C)** Effect of 6His-ArsR2 and 6His-ArsR1 proteins on *Pars1* and *Pars2* promoters, respectively. Lanes 1 to 5 were loaded with 0, 20, 50, 100, and 500 nM of 6His-ArsR2, and lanes 6 to 10 were loaded with 0, 20, 50, 100, and 200 nM of 6His-ArsR1. **(D)** Effect of As (III) and As (V) addition on the action of 6His-ArsR2 and 6His-ArsR1 on *Pars1* and *Pars2* promoters. Lanes 1 and 7 were loaded without any protein. Lanes 2 to 6 and 8 to 12 were performed with 100 nM of 6His-ArsR1 and 500 nM of 6His-ArsR2, respectively. Lanes 2 to 5 and 8 to 11 were performed with 0, 0.1, 0.5, and 2 mM of arsenite (AsIII), respectively. Lanes 6 and 12 correspond to samples with 2 mM of arsenate (AsV).

To document the role of the repressors on their cognate promoters, we performed *in vitro* transcription assays using purified 6His-ArsR1 and 6His-ArsR2 proteins, the *P. putida* RNAP (Johansson et al., 2008; the kind gift of C. Álvarez). To this end, the DNA regions of interest were assembled in plasmids pJCD-*Pars1* and pJCD-*Pars2*, which bear the corresponding sequences in a supercoiled DNA template. As shown in Figure 6A, production of 206 and 234 nt transcripts expected from the activity of the *Pars1* and the *Pars2* promoters was inhibited by increasing concentrations of the 6His-ArsR1 and 6His-ArsR2 proteins in the transcription mixtures. Moreover, these experiments demonstrated that both promoters *Pars1* and *Pars2* are constitutive in absence of regulators ArsR1 and ArsR2, but they become altogether shut down at 100 nM of ArsR1 in the case of *Pars1* and 500 nM of ArsR2 in the case of *Pars2*. To the best of our knowledge, although the repressive outcome of regulators of the SmtB/ArsR family has been shown in various ways (Busenlehner et al., 2003) this is the first time the effect has been recreated in an *in vitro* transcription assay. These results thus unequivocally demonstrate that ArsR1 and ArsR2 suffice to account for the repression of the *ars1* and *ars2* operons. The next obvious question is how arsenite comes into play.

### Effect of Arsenite on the ArsR1 or ArsR2-Mediated Repression of the *Pars1* and *Pars2* Promoters

As suggested in previous reports (Fernández et al., 2014, 2016; Páez-Espino et al., 2015; Figure 3) and taking into account the analysis of the ArsR structure, binding of arsenite to ArsR1 or ArsR2 repressors renders regulators unable to inhibit the activity of the *Pars1* or *Pars2* promoters, respectively, allowing the expression of the respective operons. Yet, although ArsR-mediated arsenite-dependent regulation has been thoroughly documented *in vivo*, there has been thus far no proof of the same *in vitro.* To address this issue, we run the same *in vitro* transcription assay explained before (Figure 6A) but adding arsenite or arsenate to the transcription mixture. As displayed in Figure 6B, addition of increasing concentrations (0.1–1 mM) of arsenite restored the appearance of the 206 or 234-nucleotide transcripts from promoter-containing DNA templates that were otherwise repressed by the ArsR proteins (Figure 6B, lanes 3–5 and 9–11). This demonstrated activation of the *Pars1* or *Pars2* promoters, respectively, and lifting of the repression caused by 6His-ArsR1 and 6His-ArsR2 on their respective promoters. However, when the same *in vitro* transcription experiment was run in presence of 2 mM arsenate (instead of arsenite), repression could not be alleviated (Figure 6B, lanes 6 and 12). These data confirm the mechanism of action of ArsR1 or ArsR2 on their respective promoters as the result of a conformational change upon specific binding of trivalent arsenite that releases the proteins from their cognate operators (Fernández et al., 2016).

### Transcriptional Cross Talk Between Both Arsenic-Responsive Promoters of *P. putida*

As mentioned above, the DNA sequences of the *Pars1* and *Pars2* promoters and their operators for the ArsR proteins are very similar (Figure 5C). Furthermore, both regulators repress their respective promoters at concentration levels that do not differ significantly in either the *in vivo* tests (Figure 3) or the *in vitro* assays (Figures 6A,B). We thus entertained a possible transcriptional cross-regulation between both arsenic detoxification operons. In order to explore whether ArsR1 and ArsR2 were able to cross-regulate the *Pars1* and *Pars2* promoters, we run the same *in vitro* transcription assay as before but mixing each promoter with the other’s regulator in the absence or presence of arsenite. The results are shown in Figures 6C,D. ArsR2 regulator was able to repress the *Pars1* promoter with similar efficiency to that shown with its cognate *Pars2* promoter, showing a total repression at a concentration of 500 nM (Figure 6C, lanes 1–5). On the other hand, ArsR1 was able to repress *Pars2* promoter in a concentration-dependent manner and with a similar efficiency than that observed with the *Pars1* promoter showing a total repression at a concentration of 100 nM protein (Figure 6C, lanes 6–10). When the *in vitro* transcription assays were repeated in presence of arsenite (0.1–2 mM), ArsR1 and ArsR2 alleviated their repression on their opposite promoter, thereby allowing activity of *Pars2* and *Pars1*, respectively (Figure 6D). These data indicated that ArsR1 and ArsR2 could efficiently bind and regulate indistinctively both *Pars1* and *Pars2*. Yet, the repression parameters of each repressor with their matching promoters were not identical. *Pars2* was totally repressed at a 100 nM of ArsR1, while it was necessary to reach 500 nM of ArsR2 to obtain a similar repression level. The situation with the promoter *Pars1* was similar; 100 nM of ArsR1 is enough to obtain a total repression while a higher 500 nM of ArsR2 was needed to get a total repression of the non-cognate promoter. It thus appears that ArsR1 binds with more efficiency to the consensus *ars* box in both promoters than ArsR2, at least under the conditions tested. As a consequence, ArsR1 seems to be a better repressor than ArsR2 in our test assay. Yet, since each As resistance operon has an optimum of performance at different temperatures (Páez-Espino et al., 2015), it could well happen that different environmental conditions favor the action of one repressor or the other. In any case, the data of Figures 6C,D not only shows that cross talk of the two *P. putida* systems is perfectly feasible, but they also expose an important feature of the regulatory node that rules tolerance of *P. putida* to this environmentally important oxyanion.

## Discussion

**FIGURE 7:**
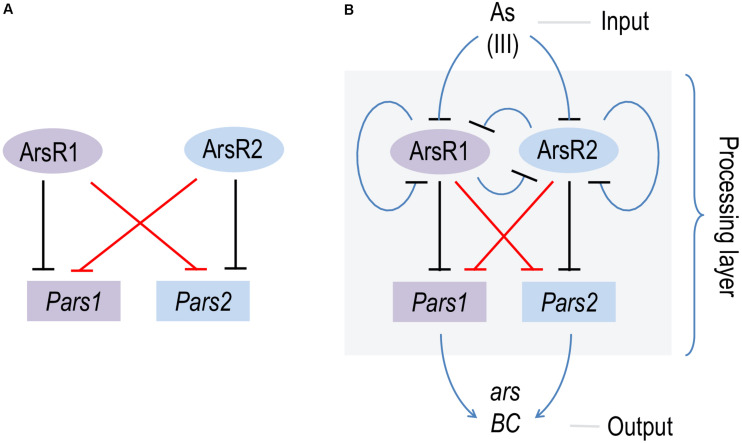
Scheme of the bifan network cross-regulation between *ars1* and *ars2* operons in *Pseudomonas putida* KT2440. **(A)** Basic scheme of the bifan network where ArsR1 and ArsR2 repress their own promoters *Pars1* and *Pars2* (black lines) and the each other (red lines). **(B)** Processing layer of the bifan network operated by direct and lateral inverter steps (blue lines) where the presence of AsIII (input) is translated in the expression of the resistance genes *arsB* and *arsC* (output).

The data above confirms *in vitro* basically all predictions on the mechanism of regulation of the two arsenic operons made earlier *in vivo* (Fernández et al., 2014; Páez-Espino et al., 2015) and with protein preparations (Fernández et al., 2016). The regulatory architecture of both *Pars1* and *Pars2* promoters is identical and somewhat odd, as it involves self-repression of the repressor (Figure 1A), an arrangement that depending on parameters often causes strong stochastic effects when the actors are in a repression mode (Alon, 2019). Although the precise K_*D*_ value of arsenite binding to the repressors could not be calculated accurately with the techniques adopted in this work, both *in vivo* and *in vitro* data suggested it was in the range of 100 μM, i.e., medium affinity. Under such conditions, the self-regulation of the *Pars* promoters by their repressors secure a steady stability of the transcriptional output (Alon, 2019). Yet, the most intriguing feature of the hereby addressed system is the coexistence of two different *ars* operons that not only run exactly the same function but can be regulated by each other’s transcriptional factors. While the maintenance of twin arsenic resistance operons has been explained both as a case of ecoparalogy (Páez-Espino et al., 2015) and/or synergistic collaboration to reach a high tolerance to the oxyanion, the efficient interplay of ArsR1 and ArsR2 with their non-cognate promoters remains puzzling. The basic scheme that represents this state of affairs is that of a bifan network motif (Figure 7; Resendis-Antonio et al., 2005), in which each regulator controls the activity of its own target as well as that of a partner in the genetic device. This type of motifs are not just casual occurrences, but they emerge as way to capturing information, integrate signals and deliver more robust control (Alon, 2019). In particular, the bifan motif provides temporal regulation of signal propagation and it synchronizes as well as filters noisy signal inputs (Lipshtat et al., 2008). As shown in Figure 7B, the basic bifan motif is operated by large number of direct and lateral inverter steps that eventually translate the presence of arsenic in the medium into expression of resistance genes proper (*arsB* and *arsC*). We thus argue that cross-regulation among the arsenic promoters and regulators is likely to shape an efficient signal-processing layer that enable survival of *P. putida* in an ample landscape of As concentrations and environmental conditions—a matter that deserves further investigations. In reality, the *P. putida* case is not an exception, as cross-regulation is a relatively common occurrence in different bacterial systems (Selin et al., 2012; Valderrama et al., 2012; Wang et al., 2013). Yet, effective cross talk between arsenic detoxification operons had not been reported before. In this respect, it is revealing that some environmental bacteria contain up to 4 ArsR variants encoded in their genome (Kang et al., 2016). That despite functional homology and high DNA sequence similarity they are kept as stably genes apart, suggest an adaptive benefit of their maintenance.

## Data Availability Statement

The original contributions presented in the study are included in the article/supplementary material, further inquiries can be directed to the corresponding author/s.

## Author Contributions

GD-R performed the experiments. DP-E constructed strains. GD-R and VdL designed the research, interpreted the data, and wrote the manuscript. All authors contributed to the article and approved the submitted version.

## Conflict of Interest

The authors declare that the research was conducted in the absence of any commercial or financial relationships that could be construed as a potential conflict of interest.

## Footnotes

1 http://blast.ncbi.nlm.nih.gov/Blast.cgi
